# Characterizing Various Posterior Fossa Tumors in Children and Adults With Diffusion-Weighted Imaging and Spectroscopy

**DOI:** 10.7759/cureus.39144

**Published:** 2023-05-17

**Authors:** Arjita Bose, Umakant Prasad, Amit Kumar, Manisha Kumari, Sanjay K Suman, Dhiraj K Sinha

**Affiliations:** 1 Radiodiagnosis, Indira Gandhi Institute of Medical Sciences, Patna, IND; 2 Radiology, Indira Gandhi Institute of Medical Sciences, Patna, IND; 3 General Surgery, Rajendra Institute of Medical Sciences, Ranchi, IND

**Keywords:** hydrocephalus, posterior fossa, tumor, magnetic resonance spectroscopy (mrs), diffusion-weighted imaging (dwi), magnetic resonance imaging (mri)

## Abstract

Background

The posterior fossa is situated between the tentorium cerebelli above and the foramen magnum below. Vital structures like the cerebellum, the pons, and the medulla are situated within it; hence, tumors within the posterior fossa are considered one of the most critical brain lesions. Children are more likely to develop posterior fossa tumors than adults. Diffusion-weighted imaging (DWI) and magnetic resonance spectroscopy (MRS) sequences along with the conventional MRI help in providing additional information in the characterization of the various posterior fossa tumors. We hereby present a series of 30 patients with clinically suspected posterior fossa masses who underwent preoperative MRI.

Objectives

This study aims to differentiate the neoplastic from non-neoplastic posterior fossa mass by evaluating the diffusion restriction pattern on DWI, quantifying the apparent diffusion coefficient (ADC) map in various posterior fossa tumors, and comparing the different metabolites of various posterior fossa tumors on MRS.

Results

Out of the 30 patients with posterior fossa lesions, 18 were males and 12 were females. Eight of them were in the pediatric age group, while twenty-two of them were adults. Metastasis was the most common posterior fossa lesion in our study sample and was found in six patients (20%), followed by vestibular schwannomas (17%) and arachnoid cysts (13%), meningiomas, medulloblastoma, and pilocytic astrocytoma (10% each) and epidermoid, ependymoma, and hemangioblastoma (7% each). The mean ADC value of benign tumors was higher than that of malignant tumors, and this difference was found to be significant (p = 0.012). The cut-off ADC value 1.21x 10^-3^mm^2^/s had a sensitivity of 81.82% and specificity of 80.47%. MRS metabolites played an additional role in differentiating benign from malignant tumors.

Conclusion

A combination of conventional MRI, DWI, ADC values, and MRS metabolites showed good diagnostic accuracy to differentiate between the various posterior fossa neoplastic tumors both in adults and children.

## Introduction

The posterior fossa is situated between the tentorium cerebelli above and the foramen magnum below. Anteriorly the clivus, anterolaterally the petrous ridge of the temporal bones, laterally the mastoid part of the temporal bones, and posterior-inferiorly the occipital bone form the bony landmark of the posterior fossa. The cerebellum, the pons, and the medulla are situated within it [1].

Children are more likely to develop posterior fossa tumors than adults. However, the distribution changes with age [2]. One of the leading causes of cancer-related deaths in the pediatric population is due to malignant tumors of the central nervous system (CNS) [3]. Age determines the location of tumors. For example, supratentorial tumors predominate in neonates, but infratentorial tumors are more prevalent in children over one year of age [4]. Among the different types of childhood brain tumors, their incidence ranges from 1 to 3 per 100,000 [5] . Childhood neoplasms occur in the infratentorial region in about 54% to 70% of pediatric brain tumors, whereas in the adult population, it is about 15% to 20% [5]. Supratentorial tumors predominate in adults. Astrocytomas and medulloblastomas are more common in the infratentorial region [6]. Common posterior fossa brain tumors in children include juvenile pilocytic astrocytoma (JPA), medulloblastoma, ependymoma, and brainstem glioma. Less frequently, atypical rhabdoid/teratoid tumor, hemangioblastoma, dermoids, schwannoma of the eight cranial nerve, cerebellar gangliocytoma, meningioma, high-grade glioma, and metastatic lesions are encountered [5]. About 15-20% of brain tumors in adults occur in the posterior fossa. Subacute strokes are the most prevalent overall lesion of the posterior fossa in adults, while vestibular schwannoma among the extra-axial and cerebellar metastases among the intra-axial lesions are the most frequent neoplastic lesions within it [1].

Symptoms occur very early with posterior fossa tumors because of their limited space. It may include drowsiness, headache, imbalance, ataxia, seizures, and symptoms associated with raised intracranial pressure like nausea, vomiting, and blurring of vision [7]. Symptoms from posterior fossa tumors also occur when the tumor damages local structures, such as the cranial nerves, or compresses the brain stem. Symptoms of cranial nerve damage include dilated pupils, facial muscle weakness, hearing loss, loss of taste, paresthesia, and vision problems [8]. Due to narrow confinement at the base of the skull, complete removal of posterior fossa tumors has certain difficulties. Therefore, accurate segmentation of posterior fossa tumors is necessary [7]. For the examination of intracranial tumors, MRI is the gold-standard imaging modality. Conventional and contrast-enhanced imaging helps us in identifying the size, shape, site, cellularity, intratumoral hemorrhage, calcification, and extension of the tumor. Compression of the surrounding vital structures and peritumoral edema can also be identified; however, it’s difficult to tell about the nature of the tumor [9].

Conventional MRI sequences are insufficient for classifying, grading the severity, or predicting therapy responses of tumors [10]. Recent studies have demonstrated the importance of other imaging modalities like diffusion-weighted imaging (DWI). DWI is an advanced imaging modality that is simple, fast, and non-invasive, with no prerequisite for contrast administration [11]. It derives extra information about tissue from the microscopic movement of the water protons [12]. Variations in water content within the tumors are due to various reasons (e.g., vasogenic edema, necrosis, hemorrhages). Apparent diffusion coefficient (ADC) values could more accurately differentiate various posterior fossa tumors by quantifying the differences in tumor cellularity [12]. In tumors, the quantification of ADC has an inverse relationship between tumor cellularity and diffusivity. DWI may assist in distinguishing tumor invasion from normal brain tissue and perilesional brain edema [13].

Additional information about the molecular nature of the tumors can be obtained with magnetic resonance spectroscopy (MRS). Proton MRS (1 H-MRS) analyzes the metabolic activity and chemical composition of the tissue studied through several major components. Long echo-time (TE) MRS shows metabolites such as choline (Cho)-containing compounds, creatine (Cr) plus phosphocreatine, N-acetylaspartate (NAA), and lactate (Lac), and short TE spectroscopy enables the detection of additional metabolites like taurine, glutamate, myoinositol (mI), or alanine (Ala) [14]. MRS metabolite ratios are used to better characterize tumor physiology [15]. Ependymal lesions and medulloblastoma are tumors with a tendency to cerebrospinal fluid (CSF) dissemination, and contrast-enhanced magnetic resonance imaging (CEMRI) of the whole spine must be performed with CEMRI brain before planning further treatment [16]. The five-year survival rates for most patients with posterior fossa tumors surpass 60% [17]. This study aims to find a noninvasive way for better characterization of posterior fossa tumors with the help of DWI with ADC and MRS.

## Materials and methods

Study design

After approval from the institutional ethics review committee, a hospital-based prospective study was conducted on 30 patients with clinical suspicion of CNS posterior fossa masses. These patients were referred to the Department of Radiodiagnosis, Indira Gandhi Institute of Medical Sciences, Patna, for an MRI of the brain from January 2021 to June 2022. Written informed consent was obtained from the patient/guardian before undergoing an MRI scan. After a detailed history and routined laboratory tests (complete blood count, hemoglobin, renal function test, and liver function test), the participants underwent plain and CEMRI of the brain. Combinations of sequences were used to classify the various types of posterior fossa tumors. All the information collected about patients during the course of the research/trial was kept strictly confidential.

Inclusion criteria: All age groups and both sexes were included.

Exclusion criteria: All non-cooperative patients, those having contraindications to MRI, patients with a history of head trauma, infections, or stroke, and patients who did not give valid consent for the study were excluded.

Sample size: The sample size was kept to a minimum of 30 patients.

Sedation: Three patients were sedated under strict clinical supervision and monitoring. Necessary emergency equipment and drugs were made available in the MRI room.

Equipment

MRI brain was done on a 1.5 Tesla superconducting MRI machine (GE made, Model Optima MR450W GEM and SIGNA Artist system) with appropriate sequences and planes. All the routine sequences were taken with patients lying in the supine position, using 5 mm slice thickness: axial T1-weighted, axial T2-weighted images, axial fluid-attenuated inversion recovery (FLAIR) images, susceptibility-weighted image, and axial diffusion-weighted images with ADC b values of 0 and 1000 s/mm2. Intravenous contrast with gadolinium (Gd-DTPA) was injected, and post-contrast T1-weighted fat-suppressed images were obtained in axial, sagittal, and coronal planes. MRS was obtained in 20 patients using both single and multi-voxel techniques, and the spectra were acquired by using a spin echo sequence (point-resolved spectroscopy [PRESS]) with short and long TEs of 30 msec and 135 msec, respectively. Two senior radiologists and a junior radiologist prospectively analyzed the MRI images without any histopathological data.

Image interpretation

The following characteristics of a space-occupying lesion in the posterior fossa were evaluated: lesion size, location, margins, heterogeneity, calcifications, hemorrhages, MR sequence appearances, enhancement pattern, perilesional edema, intracranial mass effects, involvement of any local structures, vascular invasion, and visualized spine. The ADC values of the lesions were calculated by placing the region of interest (ROIs) over the solid, enhancing, non-necrotic, and DWI-restricted parts of a solid-enhancing lesion. In a peripherally enhancing cystic lesion, ROIs were placed in the enhancing wall of the lesions. In the case of cystic lesions with enhancing mural nodules, ROIs were placed in the enhancing mural nodule. The mean ADC value was automatically calculated by the MRI software within the ROI and expressed in units of mm2/s.

The MRS parameters were analyzed for Cho, Cr, NAA, lipid (lip), Lac, and mI. Ratios of Cho/Cr and Cho/NAA were analyzed independently.

Statistical analysis

The presentation of the categorical variables was done in the form of numbers and percentages (%). On the other hand, the quantitative data with non-normal distribution were presented as a median with 25th and 75th percentiles (interquartile range). The data normality was checked by using the Kolmogorov-Smirnov test. In the cases in which the data was not normal, we used non-parametric tests. The following statistical tests were applied to the results:

1. The association of the variables which were quantitative and not normally distributed in nature was analyzed using the Mann-Whitney U test.

2. A receiver operating characteristic curve was used to find the cut-off point, sensitivity, specificity, positive predictive value, and negative predictive value of ADC for predicting malignancy.

The data entry was done in the Microsoft Excel Spreadsheet Software (Microsoft, Washington, USA), and the final analysis was done with the use of SPSS Statistics version 25.0 (IBM Corp. Released 2017. IBM SPSS Statistics for Windows, Version 25.0. Armonk, NY: IBM Corp.). For statistical significance, a p-value of less than 0.05 was considered statistically significant.

## Results

MRI findings revealed posterior fossa space-occupying lesions in 30 patients from four years to 70 years (Table 1).

**Table 1:** Age distribution of the study subjects

Age (years)	Number of cases	Percentage
0-10	5	16.6%
11-20	4	13.3%
21-30	3	10.0%
31-40	8	26.6%
41-50	3	10.0%
51-60	4	13.3%
>60	3	10.0%

Among them, 63.33% were benign and 36.67 % were malignant in nature (Table 2).

**Table 2:** Distribution of nature of lesions in study subjects

Nature of lesion	Frequency	Percentage
Benign	19	63.33%
Malignant	11	36.67%
Total	30	100.00%

In the study, among the benign category schwannoma was the most common type, followed by meningioma, while metastasis was the most common in the malignant one in adults. In children, pilocytic astrocytoma was the most common non-malignant tumor, while medulloblastoma was the most malignant one (Table 3).

**Table 3:** The various types of posterior fossa tumors in the study subjects

Types	Number of cases	Percentage (%)
Metastasis	6	20
Schwannoma	5	17
Arachnoid cyst	4	13
Meningioma	3	10
Medulloblastoma	3	10
Pilocytic astrocytoma	3	10
Epidermoid	2	7
Ependymoma	2	7
Hemangioblastoma	2	6

Among the tumors, thirteen lesions were in the cerebellum (including the cerebellar hemisphere and vermis), eight lesions were present at the cerebellopontine angle, six lesions were seen intraventricularly within the fourth ventricle, and the remaining three were seen in other locations (Table 4). Twenty-one lesions were solid, four of them were cystic, and the remaining five were cysts with mural nodules.

**Table 4:** Location of various posterior fossa tumors

Location	Number	Percentage (%)
Cerebellum	13	43.3
Cerebellopontine angle	08	26.7
Fourth ventricle	06	20.0
Others	03	10.0

Overlapping of clinical symptoms was noted in many patients. Most of them presented with cerebellar symptoms (about 87%), followed by headache (83.3%), vomiting (76%), seizures (40%), vision abnormalities (27%), hearing loss (20%), and brain stem symptoms (13%) (Table 5).

**Table 5:** Spectrum of symptoms presented by the study subjects

Symptoms	Number	Percentage (%)
Cerebellar symptoms	26	87
Headache	25	83
Nausea/vomiting	23	76
Seizure	12	40
Vision abnormality	8	27
Hearing loss	6	20
Others	4	13

DWI was done for all patients, in which 13 lesions were hyper-intense, 10 were isointense, and seven showed no diffusion restriction (Table 6). Hyperintensity on DWI was more in malignant lesions. The purely cystic lesion did not show any restrictions. Some malignant lesions showed mixed signals due to necrosis, hemorrhage, or calcifications. Cystic lesions with solid nodules showed restricted diffusion within the solid part.

**Table 6:** Distribution of DWI of study subjects DWI: diffusion-weighted imaging

DWI	Frequency	Percentage
Hyperintense	13	43.33%
Hypointense	7	23.33%
Isointense	10	33.33%
Total	30	100.00%

The mean ADC value of benign tumors was 1.76 ± 0.23 × 10−3 mm2/s and malignant tumors was 0.75 ± 0.3 × 10−3mm2/s with a statistical significance of p-value < 0.0001 (Table 7).

**Table 7:** Association of ADC X 10¯³ mm²/s with benign and malignant

ADC X 10¯³ mm²/s	Benign (n=19)	Malignant (n=11)	Total	p-value
Median (25th-75th percentile)	1.76 (1.53-3.11)	0.75 (0.71-1.185)	1.63 (0.98-1.89)	0.0001^*^

With the receiver operating characteristic curve, the cutoff for the mean ADC value for malignant lesions came to be ≤1.21 x 10−3 mm2/s with a sensitivity of 81.82% and specificity of 89.47%​ (Table 8).

**Table 8:** Receiver operating characteristic curve of ADC for predicting malignancy ROC: receiver operating characteristic curve, ADC: apparent diffusion coefficient, PPV: positive predictive value, NPV: negative predictive value

Malignancy	ADC (mm²/s)
Area under the ROC curve (AUC)	0.923
Standard error	0.0476
95% CI	0.766 to 0.988
p-value	<0.0001
Cut-off	≤1.21
Sensitivity (95% CI)	81.82% (48.2-97.7%)
Specificity (95% CI)	89.47% (66.9-98.7%)
PPV (95% CI)	81.8% (48.2-97.7%)
NPV (95% CI)	89.5% (66.9-98.7%)
Diagnostic accuracy	86.67%

MRS was further performed on a few solid tumors and cysts with mural nodules to further characterize the lesion and modify the treatment accordingly. Cerebellar metastasis, ependymoma, and medulloblastoma showed increased Cho peaks. Cerebellar pilocytic astrocytomas and hemangioblastoma showed minimally raised Cho peak with a low Cho/Cr ratio. A lip peak was seen in hemangioblastoma. An Ala peak was seen in meningioma. Vestibular schwannoma showed an mI peak, while a Lac peak was seen in the epidermoid (Table 9).

**Table 9:** MRS peaks in some posterior fossa tumors Cho: choline, Cr: creatinine, Ala: alanine, Lac: lactate, lip: lipid, mI: myoinositol

Tumor types	Metabolite peak	Chemical shift (ppm)
Metastasis	Cho/Cr	4.4
Schwannoma	mI	3.3
Meningioma	Ala	3.9
Medulloblastoma	Cho/Cr	5.3
Pilocytic astrocytoma	Cho/Cr	1.2
Epidermoid	Lac	1.3
Ependymoma	Cho/Cr	5.8
Hemangioblastoma	lip	1.3

## Discussion

MRI is preferred for the visualization of posterior fossa tumors. Diffusion-weighted MR imaging with ADC values provided additional information about tumor grades and tumor types, as well as differentiating tumors from other brain space-occupying lesions. 1 H-MRS analyzes the metabolic activity and chemical composition of the tissue to characterize specific tumor types [11]. Metastasis was the most common overall as well as intra-axial adult malignant posterior fossa lesion, while vestibular schwannoma was the most common benign extra-axial tumor, which corresponded with the findings of Tamilchelvan et al. [18]. In the present study, equal cases of medulloblastoma and pilocytic astrocytoma were present, with medulloblastoma being more malignant.

Our study comprised 30 patients. They were divided into two groups based on their age: pediatric and adult.

Among the eight cases in the pediatric population, three cases were each of JPA and medulloblastoma and two cases were of ependymoma. A substantial difference in the ADC values between these tumors was noted in our study which was similar to a study conducted by Mustafa et al. [11]. In this study, the mean ADC value was 1.67 + 0.30, 1.06 + 0.36, 0.68 + 0.22 for JPA, ependymoma, and medulloblastoma. These results were agreed with Zitouni et al. [19] who found the mean ADC ratios were 1.95 ± 0.30, 1.50 ± 0.20, and 1.02 ± 0.30 for JPA, ependymoma, and medulloblastoma. Rumboldt et al. [20] reported that pilocytic astrocytoma was the most common pediatric (CNS) posterior fossa tumor proved by histopathology representing 48.5% followed by ependymoma then medulloblastoma. In our study, the most common pediatric posterior fossa tumor was pilocytic astrocytoma followed by medulloblastoma and ependymoma.

The remaining 22 cases were put into the adult group among which six cases were of metastasis, which was the most common neoplastic tumor of the adult posterior fossa. This was in agreement with the study by Gillard et al. who stated cerebellar metastasis was the most common posterior fossa neoplasm [21]. In our results, there were three cases of posterior fossa meningiomas which showed variable restriction on DWIs. It was similar to the study done on 24 cases of typical cerebellar meningioma by Tantawy et al. [22]. The range for ADC in our study was 0.79 to 1.18 mm2/s which was within the range of ADC values obtained from the study. This study had five patients with acoustic schwannoma. All were located at a CP angle with an extension into the internal auditory canal. The acoustic schwannoma showed a higher value of ADC as compared to the meningioma. This was comparable to the study by Pavlisa et al. who found that the mean ADC value of CPA meningioma was lesser than the mean ADC value of CPA acoustic schwannoma [23]. Hemangioblastoma is a rare adult posterior fossa tumor. In our study, we found two cases of cerebellar hemangioblastoma. The mural nodule showed restriction on DWI with a significantly high ADC value. This was consistent with the study by Quadery et al. [24]. He indicated that hemangioblastoma had a higher mean ADC value when compared with brain metastasis, medulloblastoma, and ependymoma. Onishi et all. showed greater ADC values of hemangioblastomas than metastatic tumors at b = 1000 and b = 4000 [25].

The cystic mass lesions consisted of two cases of epidermoid tumors and four cases of arachnoid cysts. Epidermoid tumors were hypointense on T1WI, hyperintense on T2WI, and partially suppressed on FLAIR. They showed marked restricted diffusion on DWI with low ADC values. There was very little post-contrast enhancement. The arachnoid cysts showed low signal intensity on T1WI, high signal intensity on T2WI, and complete suppression in FLAIR. On DWI, there was low SI with high ADC values. It followed CSF signal intensity in all MR sequences. Epidermoid cysts showed restricted diffusion in DWI due to the high content of protein, cholesterol, and keratinaceous debris, while arachnoid cysts showed true cystic characteristics with no evidence of diffusion restriction in DWI. This is in line with the findings of the study conducted by Lai et al. [26].

According to the study by Paloma Mora et al., the most significant peak on spectroscopy was Cho in medulloblastoma and metastasis, mI in vestibular schwannoma, and lip in haemangioblastoma and metastasis [16]. Similar findings were observed in our study. Verma et al. found that among the brain tumors, meningiomas showed a distinct Ala peak but had variable sensitivity. This can be due to the partial oxidation of glutamine or converted from the increased pool of pyruvate due to inhibitions of the enzyme pyruvate kinase by l-Ala [27]. In our study, meningiomas showed an Ala peak.

Darweesh et al. and Moller-Hartmann et al. found additional value in the combination of MRS and ADC mapping over conventional MR sequences in differentiating and grading of brain tumors [28,29]. In our study, we found similar observations where the use of DWI, ADC, and MRS increased the diagnostic accuracy of posterior fossa tumors.

**Figure 1:**
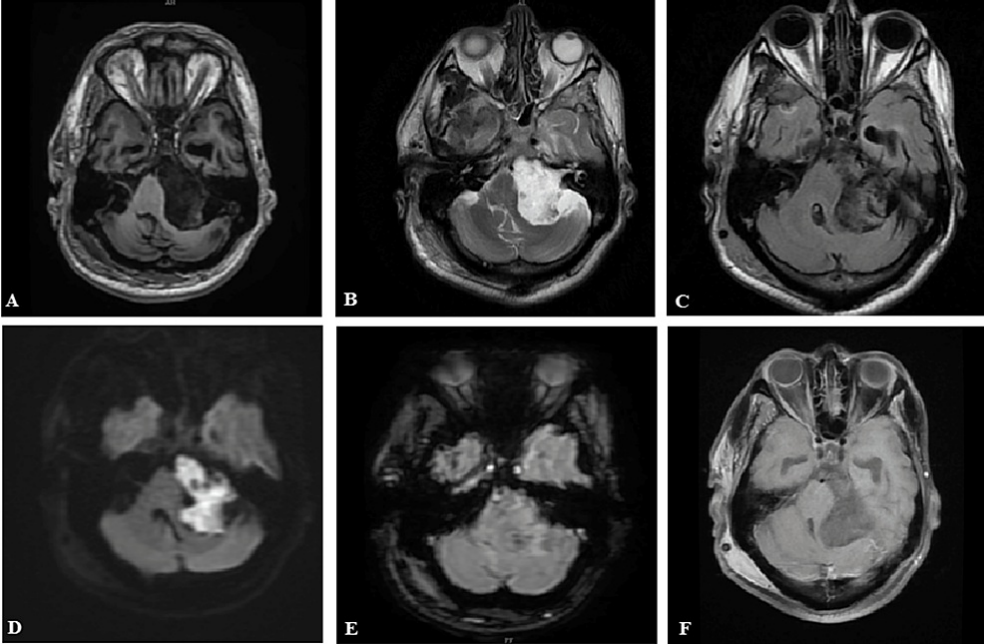
(A-F) Left cerebellopontine angle epidermoid cyst

Figure 1A-1F shows the left cerebellopontine angle epidermoid cyst. The lesion is T1 hypointense, T2 hyperintense, partial suppression on FLAIR, marked diffusion restriction in DWI, no blooming in SWAN, and no contrast enhancement.

**Figure 2:**
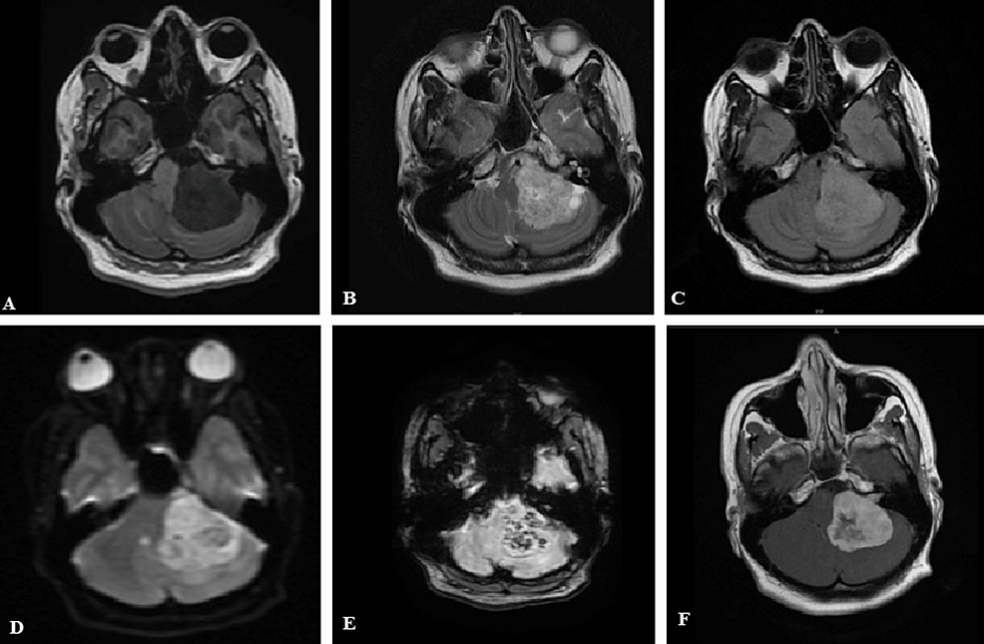
(A-F) Left vestibular schwannoma

Figure 2A-2F shows the left cerebellopontine angle vestibular schwannoma with internal auditory canal extension. The lesion is T1 hypointense, T2/FLAIR hyperintense, with patchy diffusion restriction in DWI, blooming in SWAN, and heterogeneously enhancing in the post-contrast T1 study.

**Figure 3:**
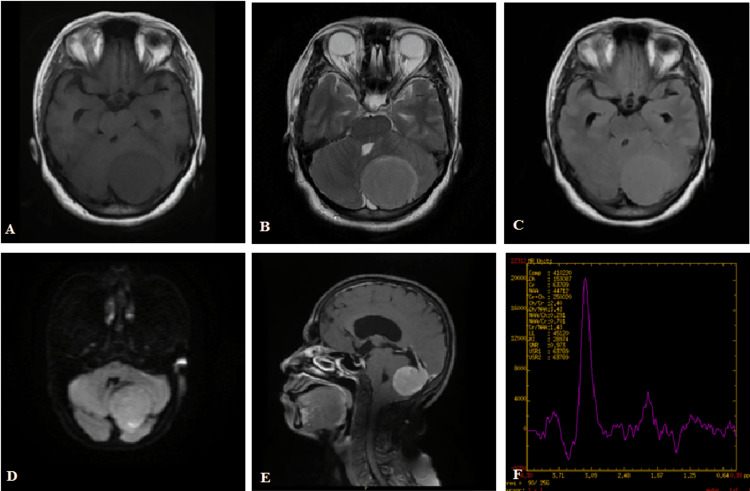
(A-F) Left tentorium meningioma with an Ala peak

Figure 3A-3F shows the left tentorium meningioma with MRS showing an Ala peak. The mass is T1 hypointense, T2 hyperintense, FLAIR isointense, patchy diffusion restriction on DWI, and homogenously enhancing with a dural tail.

**Figure 4:**
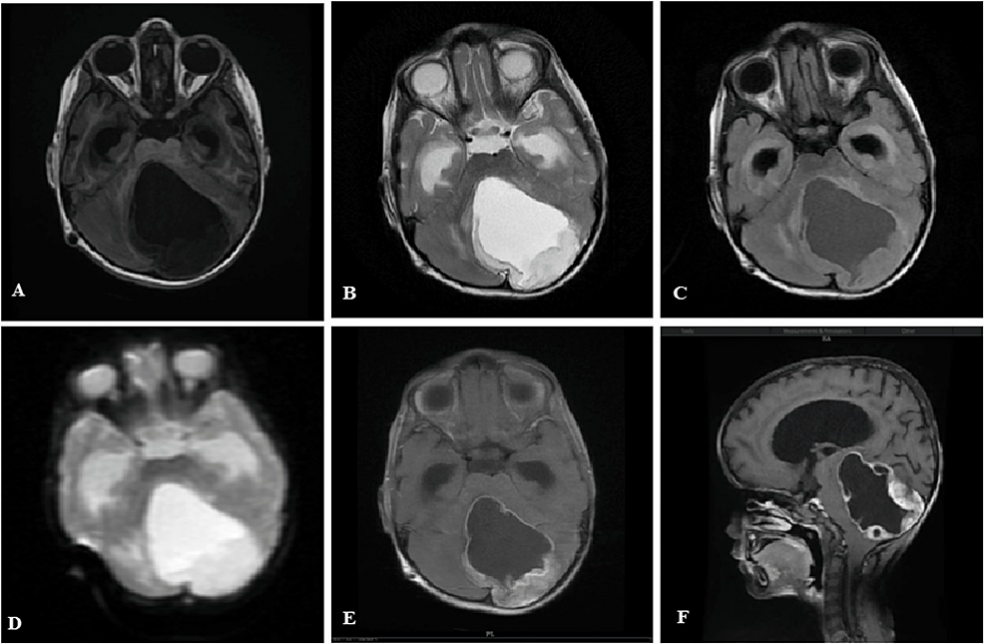
(A-F) Pilocytic astrocytoma

Figure 4A-4F shows the cerebellar pilocytic astrocytoma in a child. The lesion is cystic in nature with a mural nodule. The cystic part is T1 hypointense, T2 hyperintense, and suppressed on FLAIR (follows CSF intensity on all sequences). The wall of the cyst and the mural nodule show patchy enhancement in the post-contrast study.

**Figure 5:**
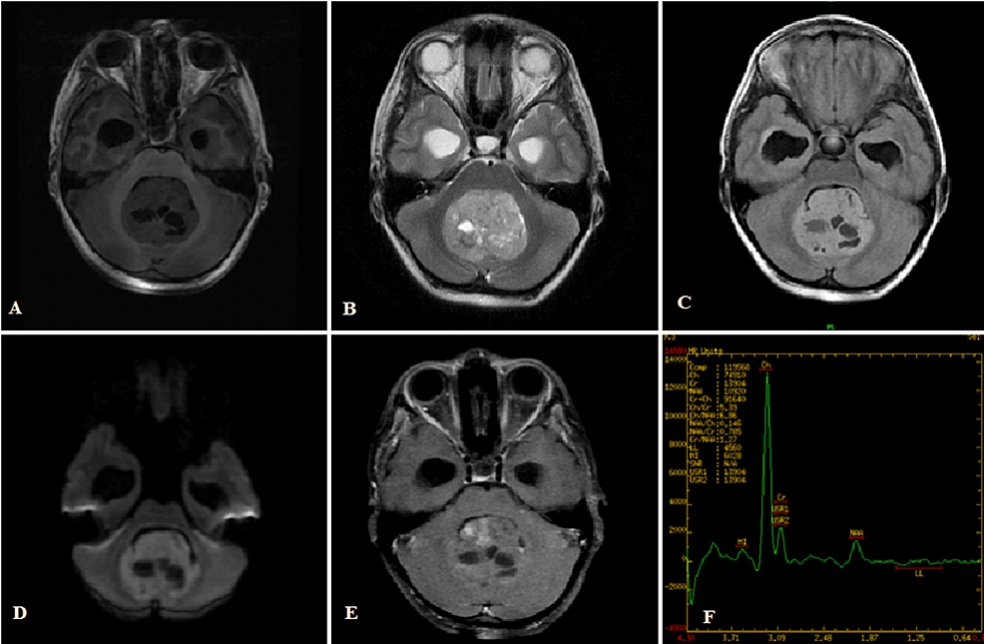
(A-F) Posterior fossa medulloblastoma with Cho/Cr peak

Figure 5A-5F shows medulloblastoma with MRS showing Cho/Cr peak. The lesion is located within the fourth ventricle which is T1 isointense, T2/FLAIR hyperintense, showing diffusion restriction, on DWI and minimal patchy enhancement in the post-contrast image. There is associated hydrocephalus.

**Figure 6:**
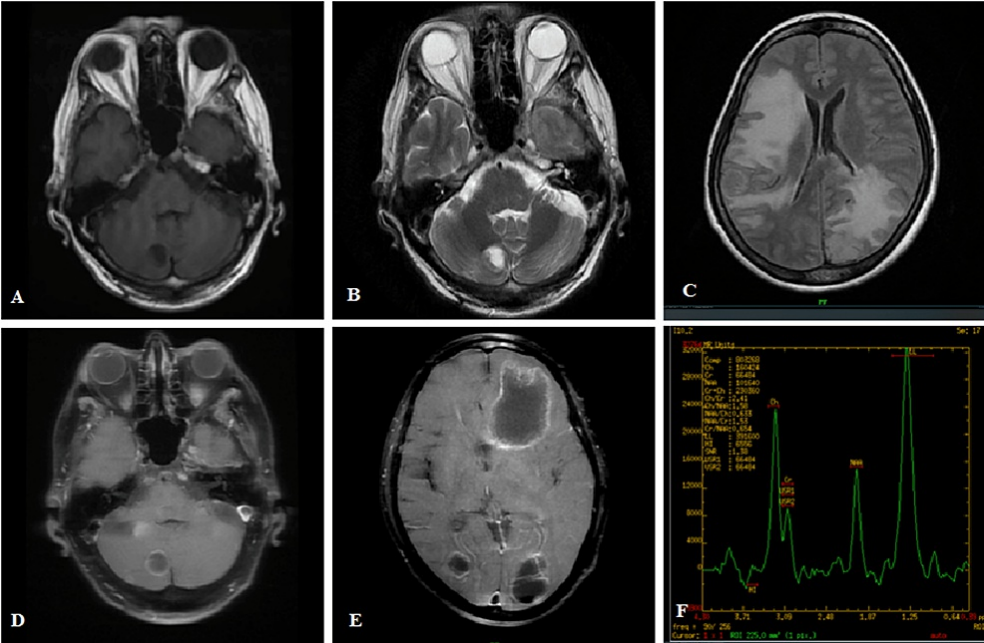
(A-F) Cerebral and cerebellar metastasis. The figure shows multiple ring-enhancing lesions with raised Cho and lip Lac peaks in a patient of known lung primary

Figure 6A-6F shows cerebral and cerebellar metastasis. The figure shows multiple ring-enhancing lesions with raised Cho and lip Lac peaks in a patient of known lung primary. There is marked perilesional edema.

**Figure 7:**
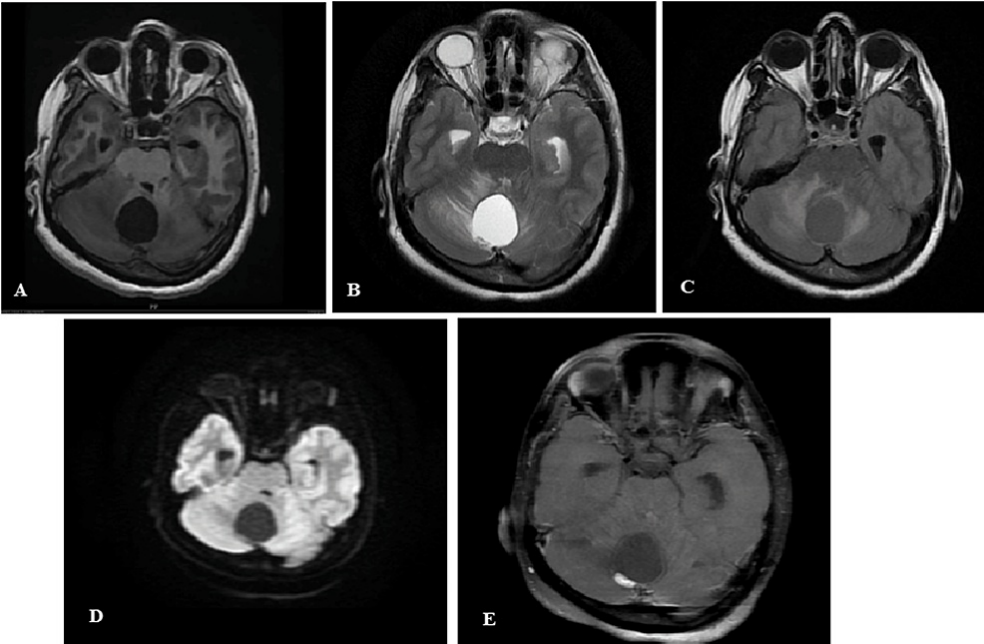
(A-E) Posterior fossa hemangioblastoma

Figure 7A-7E shows cerebellar hemangioblastoma and a cystic mass lesion with a mural nodule in a middle-aged female. The cystic component follows the CSF signal in all MR sequences. The solid nodule shows patchy diffusion restriction in DWI and enhancement in the post-contrast study.

Limitation

The present study was conducted in a sample of a limited size. Thus, future studies are required with a larger sample size in order to generalize the results. The study was limited to our hospital center only. Histopathological correlation of the imaging findings was not done as some of the patients were referred to higher centers and the rest was lost to follow-up.

## Conclusions

MRI is the imaging modality of choice for better characterization and differentiation of various posterior fossa space-occupying lesions because of its superior soft-tissue resolution and multiplanar approach. DWI with ADC is a noninvasive method used in distinguishing between benign and malignant posterior fossa tumors. In addition to DWI, an MRS study increases the accuracy of the diagnostic imaging and helps us make the diagnosis much closer to the histopathological diagnosis. Therefore, we recommend including DWI, ADC, and MRS sequences as a routine study along with the conventional MR images in the evaluation of CNS posterior fossa tumors in preoperative management and in postoperative follow-up.

## Appendices

Abbreviations

MRS: magnetic resonance spectroscopy DWI: diffusion-weighted imaging ADC: apparent diffusion coefficient JPA: juvenile pilocytic astrocytoma Cho: choline Cr: creatine NAA: N-acetylaspartate Lac: lactate mI: myoinositol Ala: alanine lip: lipid 1 H-MRS: proton magnetic resonance spectroscopy TE: echo-time CEMRI: contrast-enhanced magnetic resonance imaging CNS: central nervous system FLAIR: fluid-attenuated inversion recovery ROIs: region of interests CSF: cerebrospinal fluid
